# Mechanical, Electrical, and Tensile Self-Sensing Properties of Ultra-High-Performance Concrete Enhanced with Sugarcane Bagasse Ash

**DOI:** 10.3390/ma17010082

**Published:** 2023-12-23

**Authors:** Jinkang Lian, Yulin Wang, Tengfei Fu, Said M Easa, Yan Zhou, Huawei Li

**Affiliations:** 1 College of Architecture and Civil Engineering, Wuyi University, Wuyishan 354300, China; lianjinkangk@163.com (J.L.); lihuawei3880@126.com (H.L.); 2 College of Transpiration and Civil Engineering, Fujian Agriculture and Forestry University, Fuzhou 350108, China; 3 Engineering Research Center of Prevention and Control of Geological Disasters in Northern Fujian, Fujian Province University, Wuyishan 354300, China; 4 Department of Civil Engineering, Toronto Metropolitan University, Toronto, ON M5B 2K3, Canada; seasa@torontomu.ca; 5 College of Ecology and Resource Engineering, Wuyi University, Wuyishan 354300, China; zhouyan@wuyiu.edu.cn

**Keywords:** ultra-high-performance concrete, tensile-stress sensing, electrical resistivity, fractional resistance change, carbonation temperature

## Abstract

Although sugarcane bagasse ash (SCBA) possesses favorable cementitious properties, previous research has primarily focused on improving the mechanical performance of conventional concrete- or cement-based composites. Limited attention has been given to ultra-high-performance concrete (UHPC) with SCBA, especially regarding its tensile -sensing properties. This study aimed to comprehensively evaluate the effect of SCBA on the mechanical, electrical, and tensile self-sensing properties of UHPC. The results demonstrated that incorporating SCBA below the critical concentration of 3.0 wt% enhanced the mechanical properties of UHPC. Notably, adding 3.0 wt% SCBA remarkably improved the compressive, flexural, and tensile strengths of UHPC, resulting in increases of 13.1%, 17.4%, and 20.6%, respectively. However, excessive incorporation of SCBA adversely affected the mechanical properties due to reduced workability of UHPC, increased generation of harmful voids, and a lower degree of hydration caused by the excess SCBA. Furthermore, the inclusion of SCBA influenced the electrical resistivity of UHPC, and specifically, an SCBA content of 0.3 wt% yielded the maximum electrical resistivity. Moreover, incorporating SCBA in UHPC enhanced its tensile stress-sensing performance compared to SCBA-free UHPC. Among the various SCBA contents tested, UHPC with 0.3 wt% SCBA presented the best linearity, with values of 8.8% for loading and 17.0% for unloading, respectively, which were significantly lower than those for SCBA-free UHPC, which were 14.0% and 60.0%, respectively. Additionally, UHPC with 0.9 wt% SCBA gained the lowest hysteresis and repeatability, with values of 13.3% and 5.3%, respectively, which were much lower than those for SCBA-free UHPC, which were 50% and 51.6%, respectively. The tensile stress-sensing performance of UHPC is influenced by three key aspects: the gap between adjacent conductive fillers, contact resistance, and the connectivity of the electrical network, which are subject to change due to varying stress states and SCBA concentrations. This study should aid SCBA use and promote UHPC’s practical applications.

## 1. Introduction

As society rapidly develops, structural health monitoring (SHM) is becoming an essential technique for ensuring concrete structures’ health, serviceability, reliability, durability, and safety [1,2,3]. It is also a vital approach for timely monitoring and managing accumulated damage in concrete structures [4,5,6,7,8]. The cement-based sensors use cement composites as a conductive material [9]. By measuring the electrical resistance to external pressure, the stress and strain states in concrete structures can be monitored in situ and evaluated in real-time [10]. Adding some functional fillers (e.g., steel fibers and steel slag) to the concrete reduces resistivity and improves self-sensing [11,12,13,14,15]. This kind of sensor is more economical and more durable than conventional sensors because it is made of cementitious materials [16]. Therefore, functional fillers can replace cement in conventional, high-performance, self-sensing, and recycled aggregate concretes [17].

Superior mechanical properties are essential for applying self-sensing properties. UHPC has good durability and crack resistance and therefore can be implemented in critical infrastructures [18,19,20]. However, due to its very low electrical conductivity, UHPC alone cannot be used to achieve self-sensing. Consequently, many researchers have explored the use of different types of conductive materials to investigate UHPC’s piezoresistive sensing properties under tension [21]. Moreover, some researchers evaluated UHPC’s feasibility for monitoring flexural performance [22,23,24]. Min Kyoung Kim et al. [23] demonstrated that UHPC as a matrix substantially improved the self-sensing performance of the steel-fiber-reinforced concrete. Additionally, attached electrodes exhibited longer polarization times than embedded ones. Wu et al. [24] evaluated UHPC’s mechanical properties, electrical conductivity, and tensile sensitivity. During the stretching process, the resistivity of UHPC initially decreased and then gradually increased as the tensile strain increased. Before the peak load, the sensitivity of UHPC to tensile strain increased as the steel fiber content increased from 1% to 2%. Doo-Yeol Yoo et al. [22] examined the tensile self-sensing of UHPC with steel fibers and CNTs. The results indicated minimal signal noise and significantly lower resistance in ultra-high-performance fiber-reinforced concrete (UHPFRC) containing CNTs, primarily due to improved formation of conductive pathways. However, using nanomaterials like CNTs and graphene presents challenges such as clustering, dispersion difficulties, high cost, and potential health risks.

Several researchers developed cement-based sensors using eco-friendly materials to overcome the drawbacks of nanomaterials [25]. The abundant byproduct generated from extracting juice from the sugarcane stalk is the sugarcane bagasse (SCB), making it economical as fuel for cogeneration plants [26]. Sugarcane bagasse ash (SCBA) has potential as a supplementary cementitious material (SCM) [27,28,29]. Using SCBA as an SCM in cementitious materials can genuinely reduce greenhouse gases and carbon footprint and address the sustainability issues of cement production [30,31,32,33]. For SCBA, Hernández et al. [34] stated that sugar cane straw ash, a byproduct of sugar milling, had a certain pozzolanic activity [35]. Adding SCBA to the matrix improved the mechanical properties of cementitious materials [36,37,38], decreased hydration heat [39], improved concrete durability [30,40,41], and strengthened the cementitious matrix–aggregate interface [30]. Strategies to improve the physical properties and pozzolanic activity of SCBA include calcination [42,43,44,45], sieving [46,47], grinding [48,49], and chemical treatment [50,51]. The reactive/amorphous pozzolanic oxide composition, primarily the silica content, is the critical parameter affecting the SCM’s pozzolanic activity [30,52,53,54,55,56,57]. Therefore, SCBA may improve the mechanical and durability properties of UHPC and achieve environmental and economic benefits [58]. SCBA, as a supplementary cementitious material (SCM), has been widely studied, with more than a hundred papers published on the topic [30,39], mainly focusing on conventional cement-based composites or concrete. However, research on its impact on the performance of ultra-high-performance concrete (UHPC), especially UHPC’s tensile self-sensing performance, is still limited.

This research aimed to comprehensively evaluate the mechanical, electrical, and self-sensing properties of SCBA-reinforced UHPC. Specifically, the study objectives are four-fold: (1) to determine how the processed SCBA affects various the mechanical properties of UHPC (compressive, flexural, and tensile strengths); (2) to investigate the electrical conductivity and tensile self-sensing performance of the SCBA-enhanced UHPC; (3) to analyze SCBA’s chemical composition and porous structure and its impacts on UHPC’s mechanical properties; and (4) to analyze the combined effects of SCBA and micro steel fibers on the electrical and tensile self-sensing properties using a mechanics–electricity model. Thus, this study should aid in SCBA use and promote UHPC’s practical applications. 

This study is comprised of the following: (1) In Section 2, the manufacturing process of SCBA, including its compositions and physical structure, is investigated. Subsequently, the constituent materials of UHPC and specimen-preparation techniques are introduced. Following that, the methods and procedures for testing mechanical and electrical parameters are presented. (2) In Section 3, the workability of UHPC with different SCBA contents is investigated. Furthermore, this section delves into the mechanical, electrical, and tensile stress-sensing properties of SCBA-reinforced material. (3) In Section 4, the comprehensive tensile stress-sensing model developed to analyze the mechanism behind the improvement in the electrical and tensile stress-sensing properties of UHPC resulting from adding SCBA is explained. (4) In Section 5, the conclusions drawn from the investigations conducted are presented. It summarizes the key findings and highlights their significance in the context of the research.

**Figure 1:**
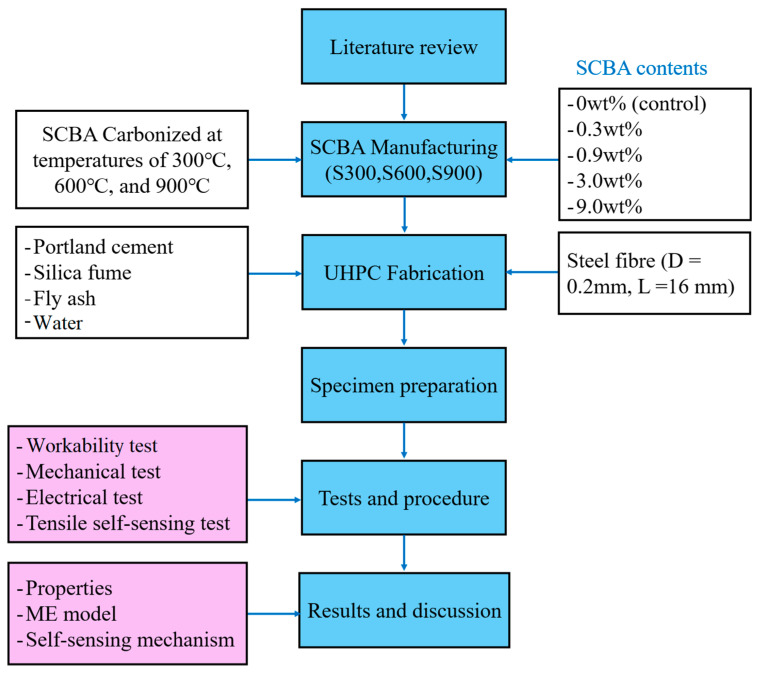
Study Routes.

Since different SCBA contents and several tests were involved, and various properties of UHPC were investigated in this research, to illustrate the procedures of this study, a block diagram of the study routes was developed to provide a clearer understanding of the structures and contents presented in this paper, and it is shown in Figure 1. The block diagram encompasses several key elements, including the SCBA manufacturing process (involving three temperatures and five contents), UHPC fabrication (involving five constituents), specimen preparation, tests and procedures (comprising four tests and their corresponding procedures), and results and discussion, covering a range of aspects such as properties, the mechanics–electricity (ME) model, and the self-sensing mechanism. The inclusion of this diagram serves to enhance the comprehension of the study’s methodology.

## 2. Experimental Program

### 2.1. Manufacturing Process of SCBA

**Figure 2:**
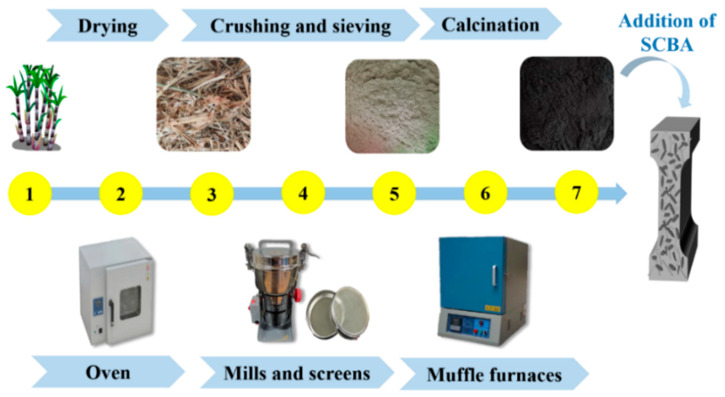
Process Flow Diagram of Sugarcane Bagasse Ash Preparation (Note: 1—Sugarcane stalk, 2—Oven drying, 3—Dried sugarcane bagasse, 4—Mill crushing and screen sieving, 5—Sugarcane bagasse powder, 6—Muffle furnace calcination, 7—Sugarcane bagasse ash, i.e., SCBA).

As reported in previous research findings [29], further processing of SCBA is required to improve its pozzolanic ability. This could increase (amorphous) biosilica content and fineness while decreasing impurities. It has been recommended to sieve, re-calcinate, and grind the SCBA as needed [30]. The specific process flow diagram of SCBA preparation is shown in Figure 2.

Table 1 and Table 2 provide information on the elemental contents, residual rate of calcinated ash and carbonation degree, and pore parameters of the porous structure of SCBA attained from different calcination temperatures. Notably, the calcination temperature significantly affects the chemical compositions and physical structure of SCBA. The chemical elements (e.g., carbon, C; hydrogen, H; and nitrogen, N), residual rate of calcinated ash, and carbonation degree decrease as the carbonation temperature increases. Among the three groups of SCBA, the sample attained at 300 °C has the lowest cumulative volume and specific surface area, but it has the largest average pore diameter (11.8 times greater than that of SCBA achieved at 600 °C). In contrast, the sample attained at 600 °C has the largest cumulative volume and specific surface area (about 4 times and 500 times greater than that of SCBA achieved at 300 °C, respectively) and possesses the smallest average pore diameter). 

**Figure 3:**
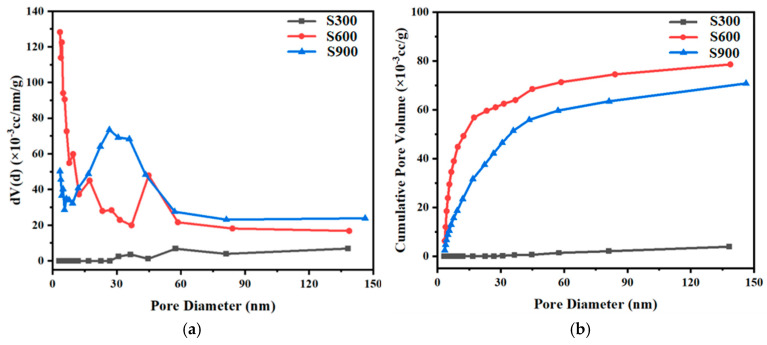
Pore distribution curves of SCBA (carbonized at 300 °C, 600 °C, and 900 °C): (**a**) primary differential curve of the cumulative pore volume and (**b**) cumulative pore volume.

The results of the liquid-nitrogen adsorption experiments in Figure 3 demonstrate that SCBA obtained at different temperatures exhibits a significant difference in pore size distribution and cumulative pore volume in size range from 0 to 150 nm. Furthermore, it reconfirms that SCBA attained at 300 °C has the smallest cumulative volume among the three groups of SCBA, while that of SCBA achieved at 600 °C is the largest.

### 2.2. Constituent Material

For UHPC fabrication, ordinary Portland cement (P.O52.5), silica fume, fly ash, and SCBA were used as binder materials, and steel fibers with a diameter of 0.2 mm and a length of 16 mm were applied as reinforcement fibers in 1.5% volume fraction of a dog-bone-shaped specimen. The chemical compositions of the binder materials are shown in Table 3. As noted, all SCBAs calcined at 300 °C, 600 °C, and 900 °C exhibit pozzolanic contents (SiO_2_ + Al_2_O_3_ + Fe_2_O_3_) greater than 70%. This meets the ASTM C618 requirements of a minimum of 50% and 70% for total pozzolanic content for class C pozzolans and for classes N and F pozzolans, respectively [59].

To evaluate SCBA’s effect on UHPC’s mechanical properties, UHPC mixtures with three kinds of SCBAs (calcined at 300 °C, 600 °C, and 900 °C) and four different contents (0.3 wt%, 0.9 wt%, 3.0 wt%, and 9.0 wt% relative to the cement) were investigated. The control group was the UHPC mixture with zero SCBA. Furthermore, considering the UHPC mixtures containing a large quantity of powder and a water-to-binder (W/B) ratio of 0.2, a polycarboxylate superplasticizer (SP) in the content of 2.0 wt% relative to the binders by weight was added to all grounds of the UHPC mixtures to improve flowability. As a result, the UHPC’s mix proportion was designed as shown in Table 4.

### 2.3. Specimen Preparation

**Figure 4:**
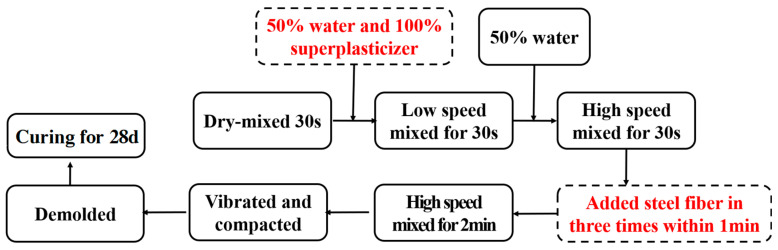
Process flow diagram of UHPC preparation.

The preparation processes of UHPC include blending mixtures, casting and compacting cementitious paste, and curing specimens. First, all the binder materials were dry-mixed for the 30 s in the mortar mixing pot, followed by adding and mixing the solution (water pre-mixed with superplasticizer) for 1 min. Steel fibers were added to the paste in three batches, and the mixtures were blended at high speed for 2 min to ensure the paste was uniform. Then, the paste was poured into the oiled molds and vibrated for 1 min on a vibrating table. After compacting the mixture, the specimens were covered with plastic film to prevent the UHPC from shrinking and cracking due to moisture evaporation. The specimens were demolded after 24 h and then underwent steam curing at a high temperature of 90 °C for 3 days to rapidly achieve ultra-high strength. Following the steam curing, the specimens were subsequently cured at an ambient temperature of 20 °C and relative humidity of 65% until testing. Figure 4 illustrates the specific process flow diagram of UHPC preparation.

### 2.4. Test Setup and Procedure

#### 2.4.1. Workability Test

The properties of workability of UHPC mixture, including fluidity and consistency, were tested following the Standard for Test Methods of Ultra-High-Performance Concrete (Chinese T/CECS 864-2021 [60]). The fluidity was evaluated using a cement mortar fluidity tester (Model No: NLD-3, Wuxi Jianyi Instrument & Machinery Co., Ltd., Wuxi, China), while the consistency was assessed using a pointer mortar consistency tester (Model No: SC-145, Xi’an Bohui Instrument and Meter Co., Ltd., Xi’an, China).

##### Mechanical Tests

**Figure 5:**
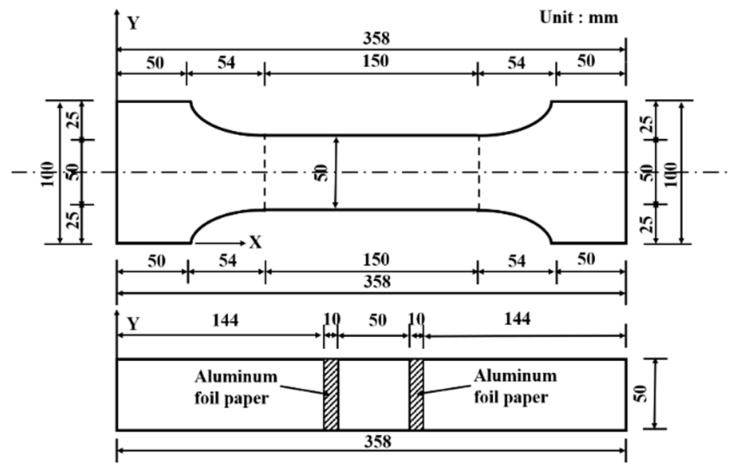
Geometrical details of the dog-bone-shaped specimens.

The mechanical properties of UHPC, including compressive, flexural, and tensile strength, were tested using the all-in-one universal testing machine (Provided by Shenzhen Wance Testing Machine Co. Ltd., Shenzhen, China, Brand: Wance, Model: HUT106D) with a maximum load capacity of 2000 KN. The Standard for Test Methods of Ultra-High-Performance Concrete (Chinese T/CECS 864-2021) was followed during strength testing. To evaluate the tensile strength, pairs of clamps were specially designed and manufactured to accommodate the dog-bone shape of specimens, as shown in Figure 5. To prevent tensile damage of the specimen at the chuck due to stress concentration during testing, the clamped area of the specimens was wrapped and reinforced by carbon fiber cloth. Subsequently, the specimen was symmetrically fixed onto the clamps of the machine, and then, tension was applied at the constant rate of 100 N/s. For the flexural strength test, a three-point bending load at a rate of 50 N/s was applied on specimens with the size of 40 mm × 40 mm × 160 mm. The compressive strength test involved the use of cubic specimens measuring 40 mm × 40 mm × 40 mm and subjected to uniaxial compression. The loading rate for the test was set at 0.6 MPa/s.

#### 2.4.2. Electrical Resistance Tests

To measure the electrical resistance of UHPC, an LCR meter was used in this study. Before formally testing the specimens, the LCR meter was calibrated to ensure the reliability and accuracy of the equipment. 

The UHPC’s electrical resistivity (ER) indicates its ability to resist transmitting ions subjected to an electrical field. The ER was measured using the two-probe method. Before measuring the specimen’s ER, a layer of silver paste was first used on the specimen’s surfaces to enhance electrical conductivity. Subsequently, aluminum foil paper was attached to the silver-paste layer (Figure 5). Two electrodes 50 mm apart were attached to the specimen. Alternating current (AC) was applied for approximately 10 min before testing to stabilize the ER of the UHPC. The ER was calculated as follows:

(1) $$\rho =R\cdot \frac{A}{L}$$

where ρ = electrical resistivity (ER) of UHPC, *R* = resistance (Ω), *A* = specimen’s cross-section area (mm^2^), and *L* = distance between the two electrodes (mm).

#### 2.4.3. Tensile Self-Sensing Tests

**Figure 6:**
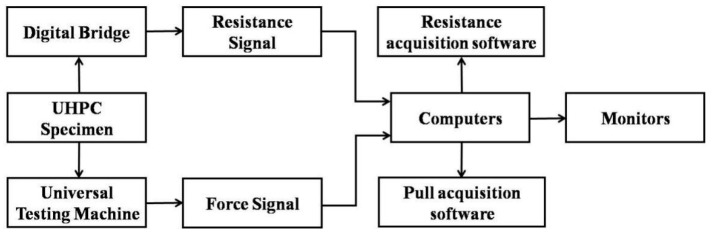
Data acquisition system.

**Figure 7:**
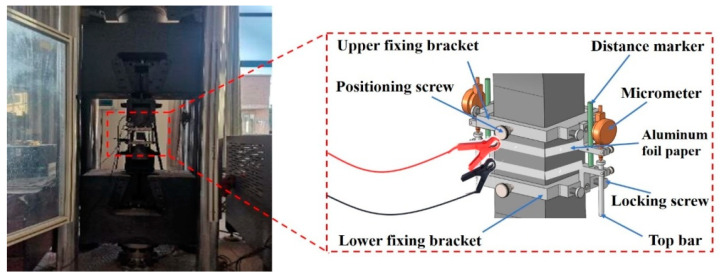
Tensile self-sensing Test.

The tensile self-sensing properties test was conducted through two kinds of loads: single-cycle tension and multi-cyclic tension with amplitude F_max_ = 7 kN (i.e., tensile stress amplitude 2.8 MPa). First, the dog-bone-shaped specimens were stretched using a UTM at 100 N/s. In addition, an LCR meter measured the electrical resistance simultaneously as the tensile force was applied, and the response was recorded at a data frequency of 10 kHz. The fractional change of resistance (FCR) was then calculated by the following:

(2) $$FCR=\frac{∆R}{R_{0}}=\frac{R_{X}-R_{0}}{R_{0}}$$

where *R*_0_ = initial resistance of the UHPC before tension application, and *R_X_* = UHPC’s resistance during tension. The specimens’ resistance and tensile stress were measured during loading using the data acquisition system. The self-sensing properties of UHPC under tension were evaluated (Figure 6 and Figure 7).

To better understand the tensile self-sensing properties, the relevant parameters (e.g., linearity, repeatability, and hysteresis error) were examined [12]. The relationship between tensile stress and the FCR of UHPC was obtained using a linear fit when a repeated load was applied. For the SCBA-UHPC, the relationship is expressed as given:

(3) $$f_{t}=a+b\;FCR$$

where $f_{t}$ = tensile stress, and *FCR* = fractional change of resistance of UHPC.

The linearity (*Lin*), the offset between the tensile stress–FCR curves, and the fitted regression line is given by the following:

(4) $$Lin=\left(\frac{∆max}{∆FCR}\right)\times 100\%$$

where ∆max = maximum deviation of tensile stress–FCR curves from the fitted regression line, and ∆FCR = FCR range.

The repeatability (*Rep*) is the FCR’s repeat degree, given by the following:

(5) $$Rep=\frac{{∆R}_{max}}{∆FCR}\times 100\%$$

where ${∆R}_{max}$ = maximum repeat difference. That is, the difference of FCR for the same tensile stress in the same stroke during repeated loading.

The hysteresis (*Hys*) is the difference in FCR concerning the same tensile stress during cyclic variation of stress and is given by the following:

(6) $$Hys=\left(\frac{{∆f}_{max}}{∆FCR}\right)\times 100\%$$

where ${∆f}_{max}$ = maximum difference in the measurement range.

## 3. Experimental Results and Discussion

### 3.1. Workability and Mechanical Properties

Table 5 illustrates the impact of SCBA on the workability of UHPC. When the same dose (3.0 wt%) was used with different calcination temperatures of SCBA, the slurry with SCBA300 had the highest fluidity of 212 mm and consistency of 81 mm. In contrast, the slurry with SCBA600 had the lowest fluidity of 191 mm and consistency of 44 mm, indicating the poorest workability of UHPC slurry due to the microstructure of SCBA achieved at 600 °C, with a specific surface area of 267.177 m^2^/g, which was much higher than that achieved at 300 °C and 900 °C (Table 2). To evaluate the effect of SCBA on the workability of UHPC mixtures, the SCBA calcinated at 600 °C was chosen for the study with different dosing levels (0.3 wt%, 0.9 wt%, 3.0 wt%, and 9.0 wt%, respectively). It was found that both the fluidity and consistency decreased as the SCBA content increased. The fluidity decreased from 209 mm to 138 mm, while the consistency decreased from 93 mm to 31 mm.

Generally, there are three main reasons for the poor workability of UHPC slurry with SCBA. Firstly, the fibrous and angular structure of the SCBA, rather than spherical particles, increases the difficulty of inflow. Secondly, the SCBA’s rough surface and irregular shape increase the friction angle of the mixture, increasing the friction of motion. Finally, the SCBA’s high specific surface area leads to high water absorption, thus reducing workability. It is evident that SCBA attained at a 600 °C calcination temperature has a much higher specific surface area than that achieved at 300 °C and 900 °C, as shown in Table 2. Therefore, the UHPC slurry with SCBA600 has the worst workability for the most addition of SCBA (9.0 wt%) attained at 600 °C.

**Figure 8:**
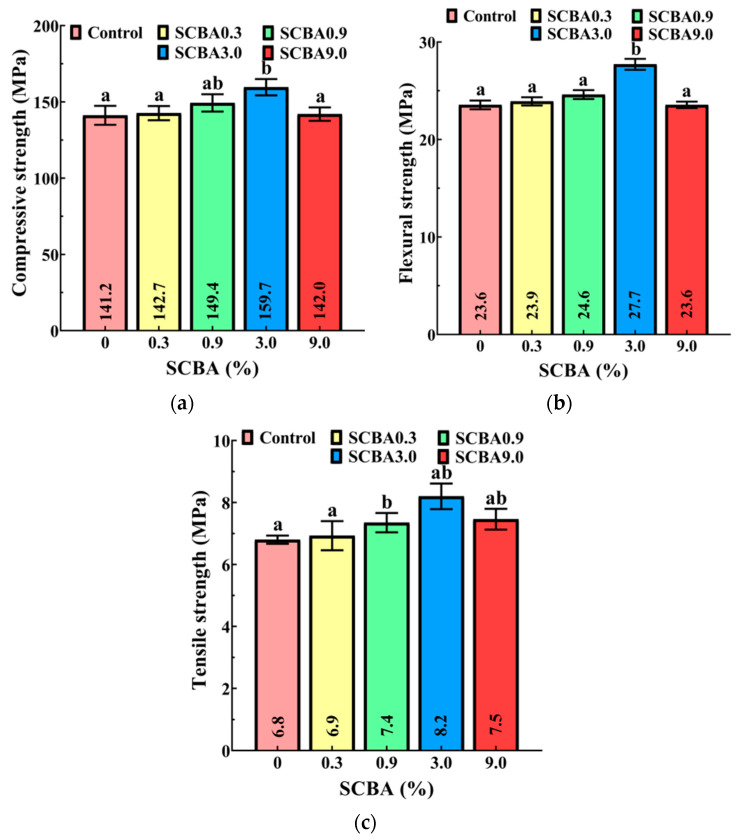
Effect of SCBA amounts on various mechanical properties: (**a**) compressive strength, (**b**) flexural strength, and (**c**) tensile strength.

The mechanical strength of SCBA with five contents of SCBA is presented in Figure 8. As observed, the compressive, flexural, and tensile strengths of the UHPC initially increased and then decreased as SCBA increased. For the control, SCBA0.3, SCBA0.9, SCBA3.0, and SCBA9.0, the compressive strength was 141.2, 142.7, 149.3, 159.7, and 142.0 MPa, respectively; the flexural strength was 23.6, 23.9, 24.6, 27.7, and 23.6 MPa, respectively; and the tensile strength was 6.8, 6.9, 7.4, 8.2, and 7.5 Mpa, respectively. The addition of 3.0 wt% SCBA resulted in the most remarkable improvement in UHPC’s mechanical properties, with an increase in the compressive, flexural, and tensile strengths by 13.1%, 17.4%, and 20.6%, respectively, relative to UHPC without SCBA. However, an excessive amount of SCBA decreased the UHPC’s mechanical properties, as adding 9.0 wt% SCBA reduced the UHPC’s compressive, flexural, and tensile strengths by 11.1%, 14.8%, and 8.5%, respectively.

The main reasons for SCBA’s effect on UHPC’s mechanical performance can be attributed to the following factors: Firstly, SCBA acts as a pozzolanic material that has high pozzolanic activity due to the amorphous silica (SiO_2_), alumina (Al_2_O_3_), and ferrite (Fe_2_O_3_), which are the principal oxides that chemically react with Portlandite calcium hydroxide during cement hydration and generate calcium silicate and aluminate hydrates. Furthermore, in the case of ground SCBA, the smaller particles contribute to the formation of additional nucleation sites for hydration products. Additionally, the use of SCBA can result in a reduction in interface thickness between the cementitious matrix and the aggregate particles, which leads to improved bulk density. Therefore, the enhanced UHPC’s mechanical properties could be attributed to the synergistic effects of these physical changes and the volcanic ash reaction. That is to say, adding processed SCBA below the critical incorporation concentration (CIC) 3.0 wt% could improve the mechanical properties of UHPC. However, incorporating an excessive amount of SCBA precisely above 3.0% could negatively impact the UHPC’s mechanical properties. This might be primarily caused by the reduced flowability and increased voids caused by the surplus SCBA within the concrete matrix.

### 3.2. Electrical Properties

**Figure 9:**
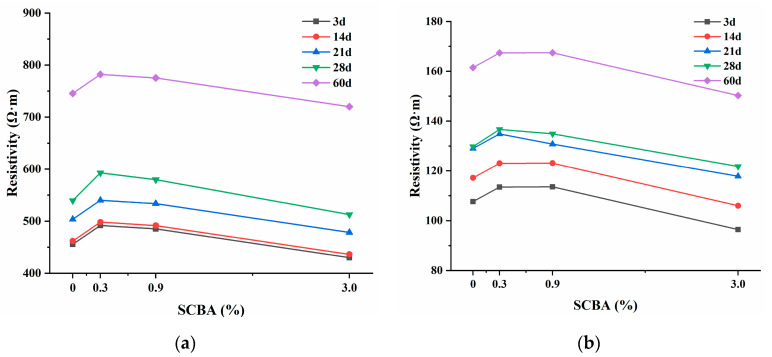
Electrical resistivity for UHPC with different SCBA contents tested at two frequencies: (**a**) 10 K Hz and (**b**) 100 K Hz.

Figure 9 illustrates the change in electrical resistivity of UHPC over 3, 14, 21, 28, and 60 days. The resistivity is affected by the curing time and decreases with increasing age due to moisture consumption during hydration. Regarding the SCBA effect, the UHPC’s electrical resistivity increases as SCBA content increases for contents less than 0.3 wt%. However, when the content exceeds 0.3 wt%, the resistivity of UHPC decreases significantly. For example, when the curing time reached 60 days, the resistivities of UHPC with four different dosages, as measured at the frequency of 100 kHz, were 161.6, 167.3, 166.4, and 150.3 Ω·m, respectively. It was observed that there was a threshold value of SCBA for resistivity. When the additional dosage of SCBA approached this threshold value of 0.3 wt%, the largest resistivity reached the maximum value. Consistent with ref. [61], the resistivity decreased with increasing frequency, but the resistivity was lower when incorporating SCBA at the same curing age and frequency.

The effect of SCBA on UHPC resistivity involves two aspects. On one side, chemical compositions of SCBA such as amorphous silica and alumina react chemically with calcium hydroxide during cement hydration and generate calcium silicate hydrates and aluminate hydrates. Consequently, the hydration products filling the gaps between conductive fillers (steel fibers and SCBA) disturb their connections, leading to higher resistivity, which is referred to as the negative effect (in respect of conductivity). On the other hand, adding SCBA reduces the proximity of adjacent conductive fillers (SCBA-SCBA, steel fiber-steel fiber, and SCBA-steel fiber) and can even create continuous conductive pathways in the matrix, particularly for high contents of SCBA, which improves the conductivity of UHPC, herein referred to as the positive effect (in respect to conductivity). Therefore, when the dosage of SCBA is less than the threshold value (<0.3 wt%), the negative effect overwhelms the positive effect, increasing the resistivity of UHPC. Conversely, when the SCBA is greater than the threshold value (>0.3 wt%), the positive effect overwhelms the negative one, decreasing the UHPC’s resistivity.

### 3.3. Tensile Self-Sensing Properties

#### 3.3.1. Single-Cyclic Tension

**Figure 10:**
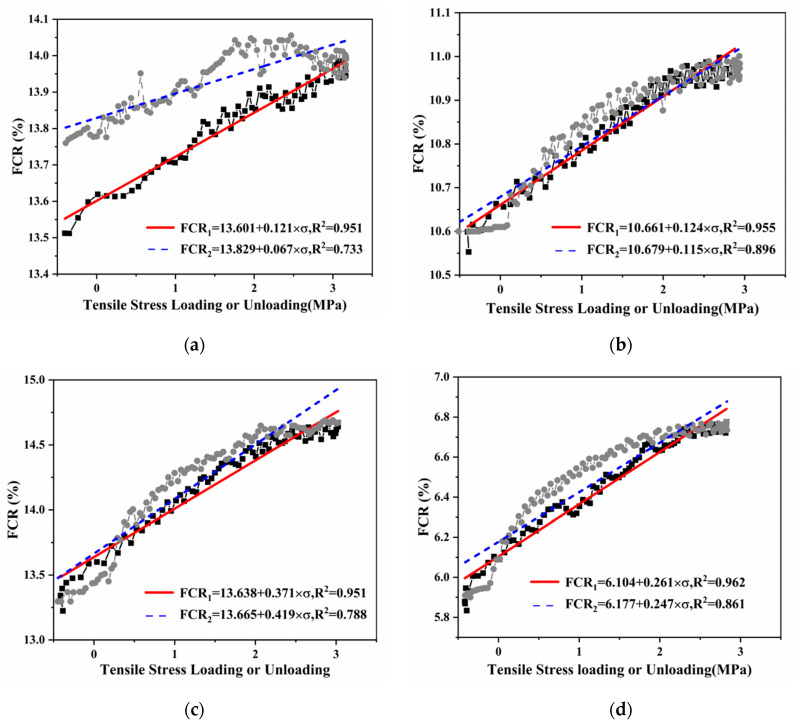
The relationship between FCR and tensile stress of UHPC with four different SCBA contents using the process of loading and unloading: (**a**) 0 wt%, (**b**) 0.3 wt%, (**c**) 0.9 wt%, and (**d**) 3.0 wt%. Note: FCR_1_ is tensile stress loading, and FCR_2_ is tensile stress unloading; ■ denotes data of loading, and ● denotes data of unloading.

To evaluate the UHPC’s tensile self-sensing properties, the relationship between FCR and tensile stress was established for the control, SCBA0.3, SCBA0.9, and SCBA3.0 (Figure 10), and the resulting tensile stress-sensing parameters are given in Table 6. The FCR of UHPC increased monotonically with increasing tensile stress during loading and decreased monotonically with decreasing tensile stress during unloading. The curve for loading is much more linear than that for unloading regardless of the SCBA content. However, UHPC with SCBA presents superior stress-sensing behavior compared to UHPC without SCBA regarding linearity, hysteresis, and sensitivity. Among the four mixtures, UHPC with 0.3 wt% SCBA showed the best linearity, with values of 8.8% for loading and 17.0% for unloading, respectively. At the same time, UHPC with 0.9 wt% SCBA exhibited the lowest hysteresis, with a value of 13.3%, which is much lower than that for UHPC without SCBA, with a value of 50%. In ref. [21], it was observed that UHPC incorporating different types of conductive powders, such as carbon black, graphite powder, nickel powder, and steel slag powder, exhibited a lower amplitude of change in FRC (all less than 4%) under a 3 MPa stress, which was significantly lower compared to the results obtained in this study.

Three factors contribute to the influence of UHPC’s self-sensing ability: the tunneling effect, contact resistance, and the connection state of the conductive network due to tensile stress. Specifically, as tension increases, the proximity between adjacent conductive fillers (i.e., SCBA–SCBA, SCBA–fiber, and fiber–fiber) increases, weakening the tunnelling effect and increasing the UHPC resistance. At the same time, both the conductive filler–matrix interface and the conductive filler–filler interface loosen, increasing contact resistance. Finally, when the tensile stress reaches a certain amplitude (about 2.8 MPa in this study) to degrade the interfaces of the conductive fillers or cause micro-damage in the matrix, the irreversibility of resistance occurs. This is why the curve for loading is much more linear than that for unloading.

Regarding the impact of SCBA, adding SCBA reduces the distances between conductive fillers, enhancing the tunnelling effect within the matrix. Thus, the resistance of UHPC with SCBA is more sensitive to changes in stress. Meanwhile, due to the presence of SCBA, the conductivity is not solely governed by steel fibers, and the UHPC maintains comparatively good conductivity even when the steel fibers are irreversibly pulled out or micro-cracks occur in the matrix due to tensile stress (about 2.8 MPa in this study). This explains the much lower hysteresis for UHPC with SCBA compared to UHPC without SCBA. However, when the additional dosage of SCBA exceeds a certain amount, continuous conductive paths will be formed in the matrix, which is irreversible if damage occurs under tension, thus changing the total current. Therefore, the hysteresis of tensile stress sensing for UHPC with SCBA above 0.9 wt% starts to worsen, although it is still better than that for UHPC without SCBA.

#### 3.3.2. Multi-Cyclic Tension

**Figure 11:**
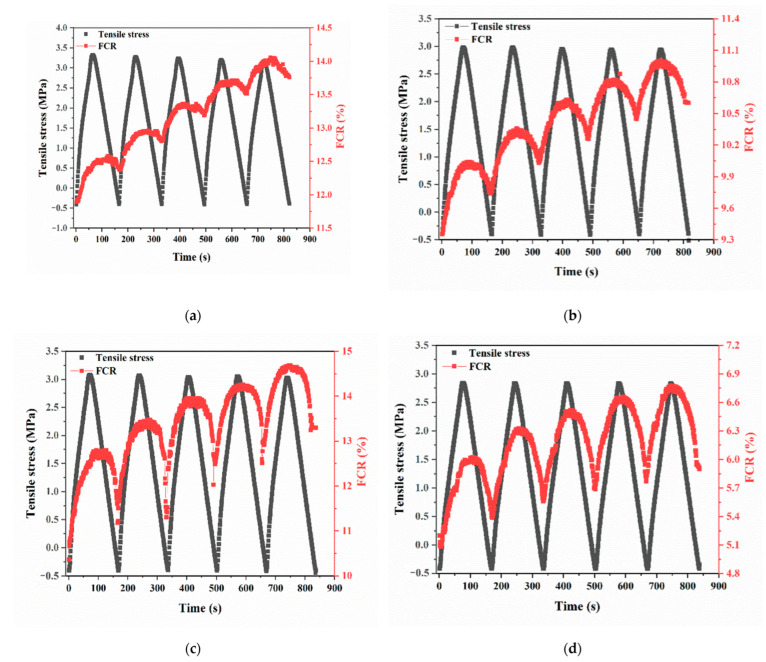
Cyclic tensile stress and FCR of UHPC with four different contents of SCBA: (**a**) 0 wt%, (**b**) 0.3 wt%, (**c**) 0.9 wt%, and (**d**) 3.0 wt%.

The relationships between tensile stress and FCR of UHPC with four different SCBA contents (0 wt%, 0.3 wt%, 0.9 wt%, and 3.0 wt%) under the multi-cyclic tensile loads are shown in Figure 11. It is evident that the UHPC resistance increases as tensile stress increases and decreases as tensile stress decreases in every cyclic load. Additionally, the FCR demonstrates an irreversible increase after each repeated tension regardless of the SCBA content. However, UHPC with SCBA exhibits superior stress-sensing capabilities in the tensile stress as compared to UHPC without SCBA due to the higher FCR amplitude (related to the sensitivity of stress sensing), higher signal-to-noise ratio, and better reversibility and repeatability. As Table 7 shows, under a tensile stress amplitude of 2.8 MPa, the FCR amplitude is 0.25%, 0.40%, 1.45%, and 0.95% for UHPC with four different SCBA contents, respectively. This indicates that stress-sensing sensitivity first increases and then decreases with the SCBA dosage, with UHPC with 0.9 wt% SCBA exhibiting the highest sensitivity. Compared with the findings in the literature [21], the FCR follows a similar trend with the tensile stress. However, in this study, the UHPC incorporating SCBA exhibited higher sensitivity. Repeatability, another crucial sensing parameter, was observed to be 51.6%, 10.7%, 5.3%, and 7.0% for composites with the four SCBA contents, respectively, showing the same trend as FCR amplitude affected by SCBA content. Among the four composites, UHPC with 0.9 wt% SCBA showed the best repeatability in terms of tensile stress-sensing characteristics. On the whole, UHPC doped with 0.9 wt% SCBA is superior in enhanced tensile stress self-sensing ability.

## 4. Tensile Self-Sensing Model and Mechanism

### 4.1. Mechanics–Electricity Model

**Figure 12:**
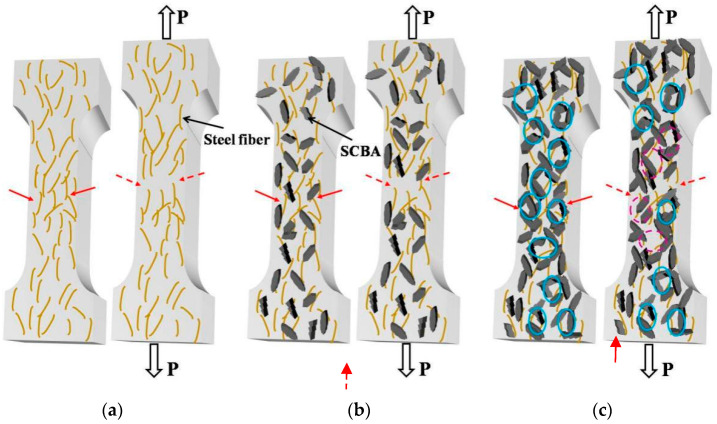
Mechanics–electricity Models for UHPC under tensile stress: (**a**) in the absence of SCBA, (**b**) with a certain amount of SCBA, and (**c**) with an excessive amount of SCBA. Note: 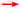 The locations where adjacent conductive fillers are connected, 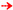 the locations where adjacent conductive fillers are disconnected due to tension, 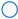 the formation of a continuous electrical network due to excessive SCBA addition, and 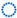 the breakage of a continuous electrical network caused by tension.

The mechanics–electrical models were established to analyze the mechanics–electrical relationship of the self-sensing UHPC, as shown in Figure 12. The models illustrate how the connection states of conductive fillers (steel fibers and SCBA) change under different levels of SCBA and applied tension. Adding SCBA to UHPC can reduce the proximity between conductive fillers and the potential barriers to charge migration within the UHPC matrix and even form continuous conductive channels, resulting in a decrease in UHPC resistance, which is beneficial to improve the self-sensing properties.

### 4.2. Self-Sensing Mechanism

The tensile stress affects UHPC resistance by changing three aspects: the gap between adjacent conductive fillers (involving the tunnelling effect), contact resistance, and the connecting state of the electrical network. According to the electron tunnelling theory, electrons within the UHPC matrix can transport between the adjacent conductive fillers, even when they are not directly connected, to generate a tunnelling current, and the spacing between the conductive fillers is the crucial factor that influences the tunnelling current. Therefore, the application of tension on the UHPC increases the gap between the adjacent conductive fillers to weaken the tunnelling effect, thus increasing the UHPC resistance. Simultaneously, increasing the tension can reduce the tightness of the interface of the conductive filler and matrix as well as the conductive filler–filler interface, resulting in increased electrical contact resistance. This elevated contact resistance makes it more challenging for electrical current to flow, leading to an increase in UHPC resistance.

When the additional dosage of SCBA exceeds a certain amount (electrical percolation value), the conductive fillers come into contact with each other, establishing a continuous electrical network. This allows electrical conduction to occur directly between conductive fillers. In this situation, the contribution degree of the tunnelling effect and interface contact with resistance change by tension is reduced, which means the FCR of UHPC is not so vulnerable to the tensile stress, and sensing sensibility gets worse. However, when the tensile stress exceeds a certain amplitude to degrade the conductive filler interfaces, pull out steel fibers, or cause micro damage in the matrix, this inevitably leads to the irreversibility of resistance; thus, the sensing behavior of UHPC will be irreversible.

## 5. Conclusions

This study evaluated the mechanical, electrical, and tensile self-sensing properties of UHPC with SCBA. SCBA’s chemical composition and porous structure were investigated to analyze their influence on the mechanical properties of UHPC. A comprehensive tensile stress-sensing model was developed to analyze the mechanism behind the improvement in electrical and tensile stress-sensing properties of UHPC that resulted from adding SCBA. Based on this study, the following conclusions are drawn:

1. Among various calcination temperatures (300 °C, 600 °C, and 900 °C), SCBA calcined at 600 °C resulted in the lowest workability of UHPC, with a fluidity of 191 mm and a consistency of 44 mm. This could be attributed to its significantly higher specific surface area of 267.177 m^2^/g, surpassing that achieved at 300 °C and 900 °C;
2. Adding processed SCBA below 3.0 wt% could improve the mechanical properties of UHPC by promoting denser hydration products such as calcium silicate and calcium aluminate hydrates. The addition of 3.0 wt% SCBA into UHPC resulted in the most significant enhancement of its compressive, flexural, and tensile strength, with improvements of 13.1%, 17.4%, and 20.6%, respectively, compared to UHPC that only contained steel fibers without SCBA. However, excessive incorporation of SCBA adversely affected the mechanical properties of UHPC. Adding 9.0 wt% SCBA degraded the compressive, flexural, and tensile strengths by 11.1%, 14.8%, and 8.5%, respectively;
3. The addition of SCBA influenced the electrical resistivity of UHPC, with a critical threshold value of 0.3 wt% for SCBA. Below this threshold, the resistivity of UHPC increased as SCBA content increased, caused by the negative effect of SCBA, wherein hydration products could disrupt the connection between conductive fillers. However, surpassing the 0.3 wt% threshold, the resistivity of UHPC decreased significantly, attributed to the positive effect, as the excess SCBA could reduce the proximity of adjacent conductive fillers;
4. UHPC containing SCBA demonstrated superior tensile stress-sensing properties compared to SCBA-free UHPC, exhibiting improved linearity and reversibility, lower hysteresis, higher sensitivity, and excellent repeatability. Among the various SCBA contents tested, UHPC with 0.3 wt% SCBA achieved the best linearity, with values of 8.8% for loading and 17.0% for unloading, respectively, which were significantly lower than those for SCBA-free UHPC, which were 14.0% and 60.0%, respectively. Additionally, UHPC with 0.9 wt% SCBA exhibited the lowest hysteresis and repeatability, with values of 13.3% and 5.3%, respectively, which were much lower than those for SCBA-free UHPC, which were 50% and 51.6%, respectively;
5. The relationship between FCR and tensile stress during loading was significantly more linear compared to unloading regardless of the SCBA contents. This could be attributed to the irreversible degradation of the interfaces between the conductive fillers or the emergence of micro-damages in the matrix during loading;
6. Overall, an SCBA content of 0.9 wt% was proven to be the most effective in improving the overall performance of UHPC, including improvements in mechanical, electrical, and tensile self-sensing performance.

## Figures and Tables

**Table 1:** Elemental Contents and Carbonation Degree of SCBA Affected by Carbonation Temperature.

	C, wt%	H, wt%	N, wt%	Residual Rate of Calcinated Ash, wt%	Carbonation Degree
S300	48.60	3.54	0.81	52.95	0.87
S600	38.09	1.67	0.64	40.40	0.53
S900	35.57	1.39	0.24	37.23	0.47

Note: S300, S600, and S900 refer to SCBA that was carbonized in a Muffle furnace at temperatures of 300 °C, 600 °C, and 900 °C, respectively. C, H, and N refer to the carbon, hydrogen, and nitrogen element contents, respectively.

**Table 2:** Parameters of Porous Structure of SCBA Attained from Different Carbonation Temperature.

	Total Pore Volume ^a^ (cc/g)	Average Pore Diameter ^a^ (nm)	Specific Surface Area (m^2^/g)
S300	4.53 × 10^−3^	33.64	0.54
S600	1.90 × 10^−1^	2.84	267.18
S900	1.54 × 10^−1^	3.02	204.54

^a^ Data are for pores smaller than 150 nm in diameter.

**Table 3:** Chemical Compositions (in terms of oxides) of the Binder Materials (wt% by weight) ^a^.

Composition wt%	CaO	Al_2_O_3_	SiO_2_	Fe_2_O_3_	MgO	SO_3_	Pozzolanic Content (SiO_2_ + Al_2_O_3_ + Fe_2_O_3_)
Portland cement	18.6	5.5	18.6	3.7	3.5	2.2	27.8
Silica fume	0.8	0.9	92.3	1.5	1.5	0	94.7
Fly ash	5.6	24.2	45.1	0.9	2.5	2.1	70.2
S300	4.0	10.5	57.9	1.9	3.4	2.8	70.3
S600	3.7	10.9	71.9	2.4	3.5	1.4	85.2
S900	2.9	9.8	75.2	2.1	1.9	1.4	87.1

^a^ SCBA obtained by sieving using sieve No. 200 (75 µm) for 2 min.

**Table 4:** Mix Proportions of UHPC (g) ^a^.

Mix	Cement	Silica Fume	Fly Ash	Water	SP	Steel Fiber	SCBA
Control	1159.7	331.3	165.7	289.9	41.4	108.5	0
SCBA300	1159.7	331.3	165.7	296.0	42.3	108.5	34.8
SCBA600	1159.7	331.3	165.7	296.0	42.3	108.5	34.8
SCBA900	1159.7	331.3	165.7	296.0	42.3	108.5	34.8
SCBA0.3	1159.7	331.3	165.7	290.5	41.5	108.5	3.5
SCBA0.9	1159.7	331.3	165.7	291.7	41.7	108.5	10.4
SCBA3.0 (SCBA600)	1159.7	331.3	165.7	296.0	42.3	108.5	34.8
SCBA9.0	1159.7	331.3	165.7	308.2	44.0	108.5	104.4

^a^ SP, superplasticizer. SCBA0.3, SCBA0.9, SCBA3.0, and SCBA9.0 refer to concrete mixtures containing SCBA at contents of 0.3 wt%, 0.9 wt%, 3.0 wt%, and 9.0 wt% relative to the cement, respectively, with the SCBA being obtained from calcination at a temperature of 600 °C. SCBA3.0 has the same mix proportions as SCBA600 and was also obtained from the same temperature of 600 °C.

**Table 5:** Effect of SCBA on UHPC workability (mm).

	Control	SCBA300	SCBA0.3	SCBA0.9	SCBA 3.0 (SCBA600)	SCBA9.0	SCBA900
Fluidity	209	212	201	196	191	138	206
Consistency	93	81	85	58	44	31	57

Note: SCBA3.0 has the same mix proportions as SCBA600 and was also obtained from the same temperature of 600 ℃.

**Table 6:** Tensile Stress-sensing Parameters of UHPC with Different Dosages of SCBA under Loading and Unloading.

Sample	FCR Range (%)	Linearity for Loading Curves (%)	Linearity for Unloading Curves (%)	Hysteresis (%)
Control	13.5–14.0	14.0	60.0	50.0
SCBA0.3	10.5–11.0	8.8	17.0	18.0
SCBA0.9	13.2–14.7	16.7	20.0	13.3
SCBA3.0	5.8–6.8	18.9	21.0	19.0

**Table 7:** Tensile Stress-sensing Parameters of UHPC with Different Dosages of SCBA under Multi-Cyclic Tension.

Sample	Total FCR Range (%)	Average FCR Amplitude in One Cycle (%)	Sensitivity (%/MPa)	Repeatability (%)
Control	11.9–14.0	0.25	0.08	51.6
SCBA0.3	9.4–11.0	0.40	0.13	10.7
SCBA0.9	10.7–14.7	1.45	0.48	5.3
SCBA3.0	5.1–6.8	0.95	0.32	7.0

## Data Availability

Data are contained within the article.
